# Genome-wide polygenic risk score for type 2 diabetes in Indian population

**DOI:** 10.1038/s41598-023-38768-5

**Published:** 2023-07-18

**Authors:** Sandhya Kiran Pemmasani, Shravya Atmakuri, Anuradha Acharya

**Affiliations:** Mapmygenome India Limited, Hyderabad, India

**Keywords:** Population genetics, Data processing, Machine learning, Endocrinology

## Abstract

Genome-wide polygenic risk scores (PRS) for lifestyle disorders, like Type 2 Diabetes (T2D), are useful in identifying at-risk individuals early on in life, and to guide them towards healthier lifestyles. The current study was aimed at developing PRS for the Indian population using imputed genotype data from UK Biobank and testing the developed PRS on data from GenomegaDB of Indians living in India. 959 T2D cases and 2,818 controls were selected from Indian participants of UK Biobank to develop the PRS. Summary statistics available for South Asians, from the DIAMANTE consortium, were used to weigh genetic variants. LDpred2 algorithm was used to adjust the effect of linkage disequilibrium among the variants. The association of PRS with T2D, after adjusting for age, sex and top ten genetic principal components, was found to be very significant (AUC = 0.7953, OR = 2.9856 [95% CI: 2.7044–3.2961]). When participants were divided into four PRS quartile groups, the odds of developing T2D increased sequentially with the higher PRS groups. The highest PRS group (top 25%) showed 5.79 fold increased risk compared to the rest of the participants (75%). The PRS derived using the same set of variants was found to be significantly associated with T2D in the test dataset of 445 Indians (AUC = 0.7781, OR = 1.6656 [95%CI = 0.6127–4.5278]). Our study demonstrates a framework to derive Indian-specific PRS for T2D. The accuracy of the derived PRS shows it’s potential to be used as a prognostic metric to stratify individuals, and to recommend personalized preventive strategies.

## Introduction

Type 2 diabetes (T2D) is one of the largest health emergencies in developing countries, and is considered as an avoidable pandemic of the twenty-first century^1,2^. According to the 2021 estimates of International Diabetes Federation (IDF), China and India have the highest numbers of people with diabetes^3^. It is further estimated that by 2045 the number of people with diabetes will have increased by 46%, with highest growth in middle-income countries. Economic development, urbanization and changed food habits could be the reasons for these increased numbers. In addition to that, genetics also plays a major role in increasing the prevalence of the disease. Several studies indicate that South Asians, in particular Asian Indians, are more susceptible to insulin resistance compared to other ethnic groups^2,4,5^. Even the migrant Indians living in different parts of the world were found to have higher diabetes rates^6,7^.

Genome-wide association studies (GWAS) done so far on different populations have identified several single nucleotide polymorphisms (SNPs) associated with T2D. Odds ratios or effect sizes obtained from those studies are used to estimate the cumulative effect called polygenic risk score (PRS). It is a weighted sum of risk alleles and their estimated effect sizes^8^. Thus estimated PRS can be used to stratify the individuals into different risk groups, and to identify at-risk individuals. For the accurate estimation of PRS, effect sizes should be taken from GWAS done on the specific population under study. Due to lack of Indian-specific effect sizes, earlier research relied on European data. Recently, Mahajan et al.^9^ provided effect sizes, in terms of summary statistics, for different populations through DIAMANTE (DIAbetes Meta-ANalysis of Trans-Ethnic association studies) consortium. Their South Asian-specific summary statistics can be used to estimate PRS for the Indian population.

Before calculating a genome-wide PRS, effect sizes of SNPs are adjusted for linkage disequilibrium (LD) among them. LD is calculated by taking a reference dataset that is as close as possible to the population used to derive the summary statistics. Though large sample sizes are recommended for such a reference dataset, 1000 Genomes Phase 3 data with 489 South Asian individuals can be used for adjusting SNP effect sizes in the South Asian population. LDpred2 is a popular program to adjust effect sizes using LD reference panel, and to calculate genome-wide PRS^10^. It uses a Bayesian algorithm to estimate posterior mean effect sizes from prior effect sizes of GWAS summary statistics. The ‘auto’ option of LDpred2 does not require any validation datasets to estimate the best-performing hyper-parameters. PRS thus calculated can further be utilized to build regression models that can predict an individual’s genetic predisposition to the phenotype of interest.

In this study, we have developed genome-wide PRS of T2D for the Indian population of UK Biobank using South Asian summary statistics^11^. The developed PRS was tested on an independent dataset from GenomegaDB of Mapmygenome^12^. To our knowledge, this is the first study to systematically evaluate the utility of South Asian-specific summary statistics of T2D on the Indian population. The developed PRS can be used as a prognostic metric to identify high risk individuals early on in life, and to recommend personalized preventive measures.

## Methods

### Study participants

#### UK Biobank

UK Biobank is a large, population-based prospective study, with over 500,000 participants, aged 40–69 years when recruited in 2006–2010, living in the United Kingdom^11^. Extensive phenotypic and genotypic data of the participants was collected across four assessment visits. Data of 3,983 Indian participants (Field ID#: 20115) were used in the present study to build polygenic risk scores for T2D. Participants were excluded based on—mismatch between reported sex and genetic sex; sex chromosome aneuploidy; excessive or low heterozygosity; outliers based on 3 standard deviations from the mean of top 3 principal components; and relatedness with kinship coefficient > 0.088^13^. Diabetic cases were identified based on International Classification of Diseases (ICD) codes 9 and 10, self-report, doctor diagnosis, HbA1C levels and medication for diabetes. Data fields and codes are given in Table 1^14–16^. Individuals with type 1 diabetes (self-reported code 1222 without mention of 1223 or ICD10 code E10 without mention of E11) were excluded from the analysis. Age at diagnosis of T2D was taken as earliest of doctor diagnosed age (Field ID#: 2976), self-reported age (Field ID#: 20009), first in-patient diagnosis in ICD10 records (Field ID#: 41280), ICD9 records (Field ID#: 41281) and age at assessment of initiating medication (Field ID#: 21003). Individuals were excluded if the age at diagnosis of T2D was less than 30 years or information was not available on age at diagnosis.

**Table 1:** Selection of T2D cases from UK Biobank.

Field name	Field ID	Code
Diabetes diagnosed by a doctor	2443	1—Yes; 0—No; –1/–3/NA—Missing
Self-reported	20002	1220, 1223, 1276, 1468, 1607
HbA1c	30750	> = 48 mmol/mol; NA—Missing
Medication for cholesterol, blood pressure, diabetes, or take exogenous hormones [Female Question]	6153	3 −1/−3/NA—Missing
Medication for cholesterol, blood pressure, diabetes [Male Question]	6177	3 −1/−3/NA—Missing
Treatment/Medication	20003	1140857494, 1140857496, 1140857500,1140857502, 1140857506, 1140857584, 1140857586, 1140857590, 1140874646, 1140874650, 1140874652, 1140874658, 1140874660, 1140874664, 1140874666, 1140874674, 1140874678, 1140874680, 1140874686, 1140874690, 1140874706, 1140874712, 1140874716, 1140874718, 1140874724, 1140874726, 1140874728, 1140874732, 1140874736, 1140874740, 1140874744, 1140874746, 1140883066, 1140884600, 1140921964, 1141152590, 1141153254, 1141153262, 1141156984, 1141157284, 1141168660, 1141168668, 1141169504, 1141171508, 1141171646, 1141171652, 1141173786, 1141173882, 1141177600, 1141177606, 1141189090, 1141189094
ICD10	41270	E11–E14
ICD9	41271	250

#### GenomegaDB

GenomegaDB of Mapmygenome is a genotype and phenotype database of Indians living in India. Genotype data was generated using Illumina’s HumanCoreExome-12 (HCE-12), HumanCoreExome-24 (HCE-24) and Infinium Global Screening Array-24 (GSA-24). Phenotype data was collected through a printed questionnaire that included individual clinical history, operative procedures, medications, family history, country of birth, among others. Written informed consent, including the consent to use data for research, was taken from each individual. In the current study, samples processed on GSA-24 arrays version 1.0, 2.0 and 3.0 were considered. Standard QC on samples included—removing samples with low call rate (< 95%), gender mismatch, extreme heterozygosity, relatedness or that were outliers in principal component analysis (PCA). Diabetic cases and controls, aged more than 30 years, were selected based on self-reported clinical history and medications.

### Genotype data

#### UK Biobank

**Figure 1:**
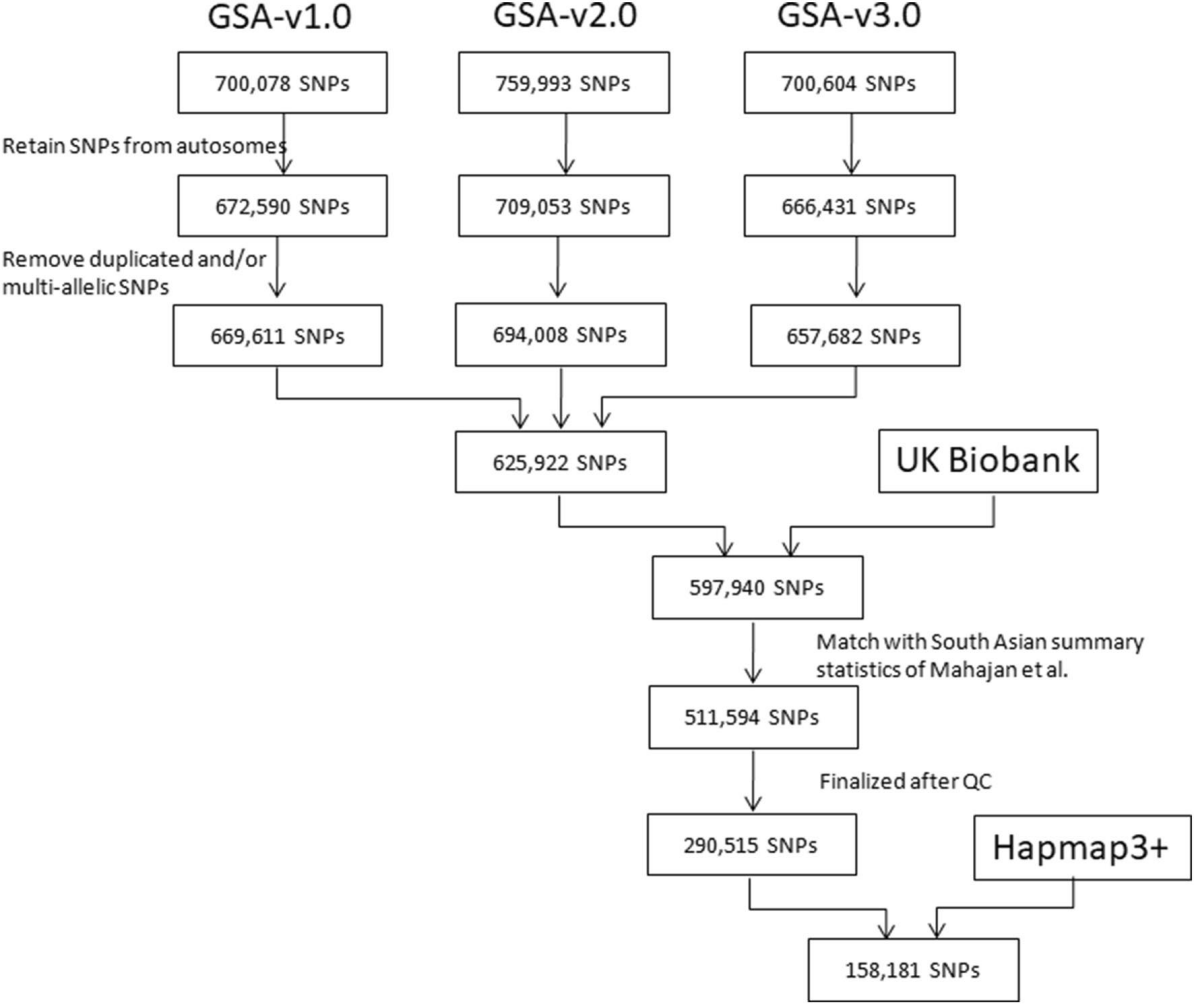
Flowchart depicting the SNP selection.

UK Biobank v3 imputed data, available in BGEN v1.2 format, was used in the analysis (Field ID#: 22,828). Only the variants that overlap with the ones present on GSA chips were considered (Fig. 1). QCTOOL v2^17^ was used to retrieve the samples and variants of interest. Further filtration was done for INFO score >  = 0.3 and minor allele frequency (MAF) >  = 0.05. MAF filter not only helps to maintain good genotyping and imputation quality^28,29^, but also to have presence of polymorphism across the datasets.

#### GenomegaDB

625,922 autosomal bi-allelic SNPs that were genotyped across the three versions of GSA chip were considered in the analysis. Genotypes were phased using SHAPEIT v2.15^18^, and missing ones were imputed with IMPUTE v2.3 software^19^, using 1000 Genomes Phase 3 data of South Asians as reference (Fig. 1).

In the calculation of PRS, we restricted the analysis to 1,444,196 Hapmap3 + variants, as recommended by authors of LDpred2^20^. 158,181 SNPs that overlapped with GSA chips, UK Biobank imputed data, South Asian summary statistics of DIAGRAM consortium and Hapmap3 + variants were considered in the analysis.

### Summary statistics

Summary statistics from South Asian-specific GWAS meta-analysis released by Mahajan et al., with 16,540 cases and 32,952 controls, were obtained from DIAMANTE consortium^9^. QC on summary statistics was done as per the method proposed by Prive et al.^21^. Effective sample size (n_eff_) was calculated as 4/((1/n_cases_) + (1/n_controls_)). Then, standard deviation of genotypes (sd_ss) was calculated as 2/sqrt(n_eff_ * beta_se^2 + beta^2)), where ‘beta’ is the effect size and ‘beta_se’ is the standard deviation of effect size. Standard deviation from the allele frequencies (sd_af) was calculated as sqrt(2*f*(1−f)), where ‘f’ is the effect allele frequency given in summary statistics. Variants were filtered out if sd_ss < (0.5 * sd_af) or sd_ss > (sd_af + 0.1) or sd_ss < 0.1 or sd_af < 0.05. Variants were also filtered out if the absolute difference in allele frequencies of the UK Biobank data and the frequencies given in summary statistics was > 0.1.

### LD reference panel

1000 Genomes Phase 3 data in PLINK format was obtained through PLINK2 resources^22^. To increase the predictive power of PRS, the South Asian panel (SAS), composed of 489 individuals, was considered.

### Polygenic risk scores (PRS)

Polygenic risk scores were generated using the LDpred2 algorithm implemented in ‘bigsnpr’ package (version 1.11.6) of R^23,24^. The ‘*auto*’ option, which directly estimates the model parameters from the data without the requirement of training data, was used along with *shrink_corr* = 0.95 and *allow_jump_sign* = FALSE, as per the procedure recommended by LDpred2 authors^25^. PRS were normalized to have mean zero and standard deviation one.

### Prediction of type 2 diabetes

To understand the association of PRS with T2D, logistic regression model was built with age, sex and top 10 principal components of genotype data as covariates. Model accuracy was assessed using standard receiver operating curves (ROC). Analyses were done with R v4.2.

## Results

Out of 4161 Indian participants of UK Biobank, genotype data was available for 3983 participants. After the initial QC, 3777 participants were included in the final analysis, of whom 959 were T2D cases and 2818 were controls. In the case of GenomegaDB, 327 cases and 396 controls were selected based on availability of genotype data. After sample QC, we were left with 194 cases and 251 controls. Table 2 gives information on characteristics of participants included in the analysis from UK Biobank and GenomegaDB. Mean age of participants of UK Biobank was higher compared to that of GenomegaDB.

**Table 2:** Characteristics of participants from UK Biobank and GenomegaDB.

	UK Biobank	GenomegaDB
Number of participants	3777	445
Number of T2D Cases, n (%)	959 (25.4%)	194 (43.6%)
Age, mean (SD)	56.7 (8.5)	48.4 (11.3)
Male, n (%)	1916 (50.7%)	294 (66.3%)

597,940 autosomal SNPs, with INFO score >  = 0.3, and overlapping with SNPs of Illumina’s GSA arrays versions 1.0, 2.0 and 3.0, were considered in the analysis. South Asian specific GWAS summary statistics obtained from the DIAMANTE consortium contains information on 10,401,621 SNPs. QC on summary statistics and UK Biobank genotype data resulted in 290,515 SNPs, out of which 158,181 Hapmap variants were finally used in developing genome-wide PRS (Fig. 1).

**Figure 2:**
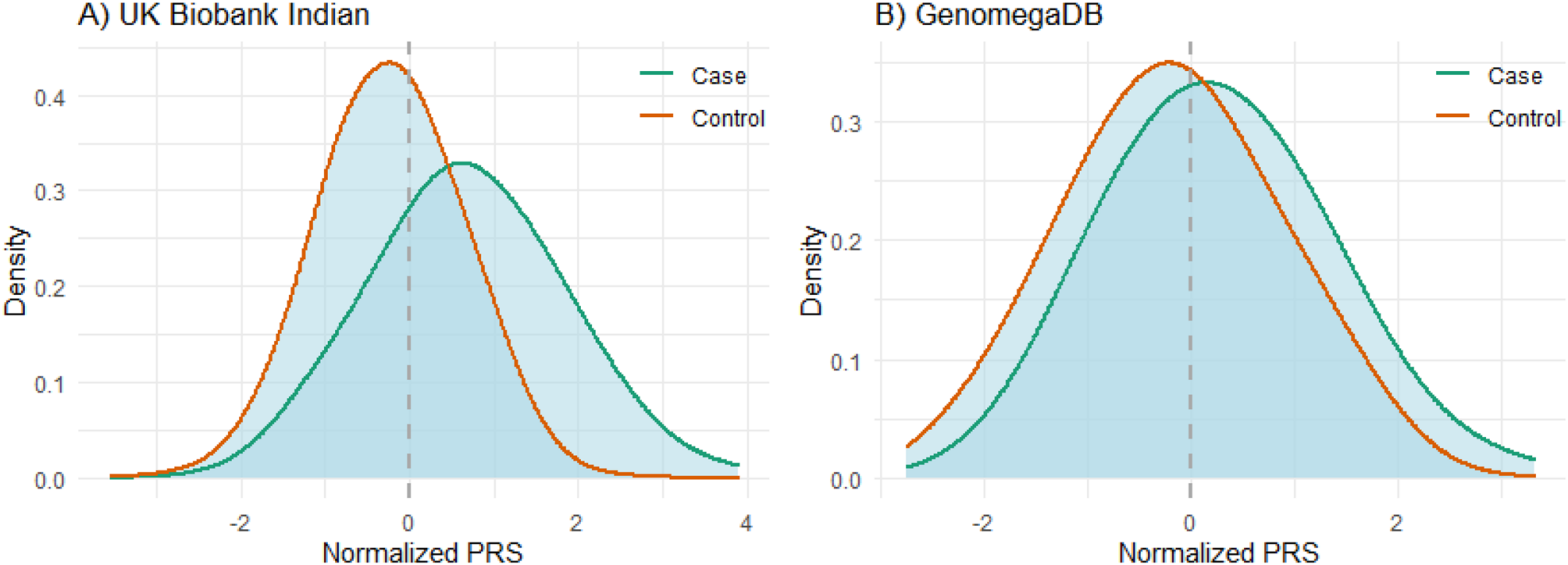
Distribution of normalized PRS. (**A**) UK Biobank Indian (**B**) GenomegaDB.

The LDpred2 algorithm, along with the South Asian 1000 Genomes LD Reference panel, was used to correct the effect sizes given in summary statistics. PRS for each sample was calculated as a sum of the number of risk alleles weighted by the adjusted effect sizes. Figure 2A shows the distribution of normalized PRS in cases and controls.

**Figure 3:**
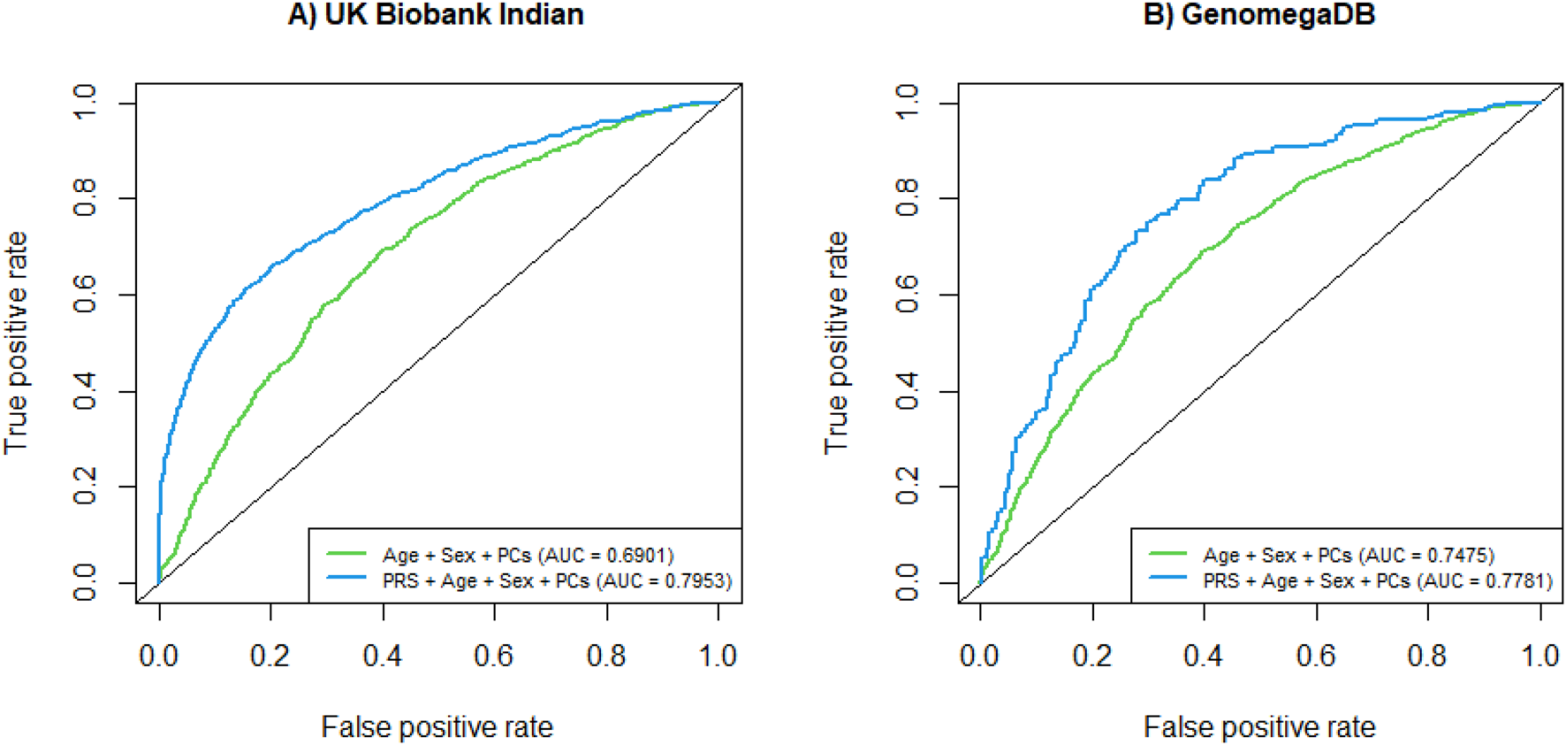
AUCs of PRS developed on data from Indian samples of UK Biobank (**A**) and GenomegaDB (**B**).

Addition of PRS to the logistic regression model with age, sex and top 10 principal components of genotypes improved the accuracy of T2D risk prediction, increasing the AUC from 0.6901 to 0.7953 (Table 3 and Fig. 3). PRS showed an adjusted odds ratio of 2.9856 (95% CI: 2.7044–3.2961). When samples were divided into PRS quartiles, and the lowest quartile was taken as reference, all sequential PRS groups showed high risk of developing T2D (Table 4). The risk of developing T2D after adjusting for age, sex and top 10 principal components of genotype data was 9.82 fold higher in the participants of the fourth quartile (top 25%) when compared with the participants of the first quartile (bottom 25%). The risk was 5.79 fold higher when the top 25% of participants were compared with the rest of 75%.

**Table 3:** Association analysis of genome-wide PRS with Type 2 Diabetes.

Dataset	OR/SD	AUC	
		Age + Sex + PCs	PRS + Age + Sex + PCs
UK Biobank Indian	2.9856	0.6901	0.7953
GenomegaDB	1.6656	0.7574	0.7781

**Table 4:** Association analysis of genome-wide PRS with T2D across different quartiles of PRS.

Reference quartile	High PRS quartile	UK Biobank Indian			GenomegaDB		
		OR/SD	95% CI	P value	OR/SD	95% CI	P value
1st quartile	2nd quartile	1.27	0.96–1.67	0.0964	1.28	0.67–2.43	0.4544
1st quartile	3rd quartile	2.84	2.18–3.69	6.27e−15	2.07	1.06–4.05	0.0327
1st quartile	4th quartile	9.82	7.56–12.75	< 2e−16	2.85	1.47–5.51	0.0019
1st, 2nd, 3rd quartiles (Bottom 75%)	4th quartile (Top 25%)	5.79	4.86–6.90	< 2e−16	2.27	1.38–3.71	0.0012

In order to test the performance of PRS in an independent dataset, 194 cases and 251 controls were selected from GenomegaDB of Mapmygenome. Figure 2B shows the distribution of normalized PRS in cases and controls of GenomegaDB. Addition of PRS to the model with age, sex and top 10 principal components of genotypes improved the accuracy of T2D risk prediction, with AUC changing from 0.7574 to 0.7781. The risk of developing T2D was 2.85 fold higher in samples of the fourth quartile (top 25%) when compared with samples of the first quartile (bottom 25%).

## Discussion

In this study, we derived genome-wide PRS of T2D for the Indian population, using Indian case–control samples available at UK Biobank. LDpred2 algorithm, with weights extracted from South Asian summary statistics of DIAMENTE consortium, gave PRS that was significantly associated with T2D (AUC: 0.7953). Participants in the fourth PRS quartile (top 25%) showed 5.79 folds increase in genetic risk compared to the rest of 75%, after adjusting for age, sex and top 10 genetic principal components. There was no significant difference in first and second quartiles. Data from GenomegaDB was used to validate the PRS, and to replicate the association. In spite of smaller sample size, the developed framework proved the significance of PRS in identifying T2D incidence (AUC: 0.7781). It showed 2.27 fold increased risk of diabetes in the top quartile (top 25%) compared to the rest of 75%. There was 2.85 fold increased risk in top quartile (top 25%) compared to bottom quartile (bottom 7%). This indicates the importance of PRS in stratifying the individuals into different risk groups.

The biggest hurdle in developing genome-wide PRS for the Indian population is lack of summary statistics for SNP associations with T2D. Predictive ability of PRS is compromised if the effect sizes and frequencies are taken from other population groups. Earlier study done by Lamri et al.^26^ on prediction of gestational diabetes in South Asian women showed that the accuracy of PRS was higher with mutli-ethnic summary statistics, which includes South Asian samples, than that of European. Similar results were observed by Hodgson et al.^27^ while constructing T2D PRS for British Pakistanis and Bangladeshis. Now, the availability of South Asian summary statistics from the DIAMENTE consortium facilitated the development of a framework for accurate estimation of PRS for the Indian population. This PRS showed superior performance compared to that of multi-ethnic and European summary statistics [in-house unpublished results].

LDpred2-auto method makes the construction of PRS an easy process compared to its counter-methods LDpred2-inf and LDpred2-grid which need validation data to estimate the hyper-parameters. The recent publication from Prive et al.^24^ gave many suggestions on improving the performance of the algorithm. Especially, quality control on summary statistics improves the predictive performance of PRS. Though LD metrics calculated from South Asian samples of 1000 Genomes project were used in this study, a bigger dataset is recommended. Restricting the analysis to Hapmap3 + variants resulted in smaller set of variants being considered, but may be justified due to the advantage it brings in stability of the analysis.

Assessment of UK Biobank participants was done at four different time points. Availability of follow-up data, along with data from different questionnaires and biochemical assays, allowed the reliable detection of diabetic patients. In the case of GenomegaDB, controls were much younger than that of UK Biobank, with a potential to become diabetic cases in future. Also, assessment of T2D status was purely based on self-report, which might result in a few misclassifications. In spite of lacking follow-up data, GenomegaDB has the advantage of coming from Indians living in India. For lifestyle disorders, like diabetes, it is preferable to take data from the native population having the same lifestyle and environment to that of the population for which inferences are made. Also, the present study included age, sex and PRS as risk factors in developing predictive models for T2D. But including the other clinical and lifestyle variables, such as BMI, HDL, LDL, physical activity, sleep duration, smoking and alcohol consumption will improve the prediction accuracy of the models.

In conclusion, Indian-specific PRS developed by us showed high accuracy in predicting the risk of developing T2D. Results from UK Biobank and GenomegaDB datasets indicated that genome-wide PRS holds strong potential to be adopted in clinical care to identify high risk individuals and in early intervention to guide towards healthier lifestyles.

## Data Availability

This research has been conducted using the UK Biobank Resource under Application Number 81481. UK Biobank data is available to researchers by registration through https://www.ukbiobank.ac.uk/enable-your-research/register. GenomegaDB is available on research collaboration with Mapmygenome India Limited by contacting anu@mapmygenome.in.
